# Wnt/β-catenin signal transduction pathway in prostate cancer and associated drug resistance

**DOI:** 10.1007/s12672-021-00433-6

**Published:** 2021-10-10

**Authors:** Chunyang Wang, Qi Chen, Huachao Xu

**Affiliations:** 1 Urology Department, PLA General Hospital, Beijing, 100853 China; 2 Department of Medical Oncology, The First Affiliated Hospital of USTC, Division of Life Sciences and Medicine, University of Science and Technology of China, Hefei, 230031 Anhui China; 3 Department of Urologic Oncology Surgery, The First Affiliated Hospital of USTC, Division of Life Sciences and Medicine, University of Science and Technology of China, Hefei, 230031 Anhui China

**Keywords:** Wnt/β-catenin pathway, Androgen receptor, Prostate cancer, Drug resistance

## Abstract

Globally, prostate cancer ranks second in cancer burden of the men. It occurs more frequently in black men compared to white or Asian men. Usually, high rates exist for men aged 60 and above. In this review, we focus on the Wnt/β-catenin signal transduction pathway in prostate cancer since many studies have reported that β-catenin can function as an oncogene and is important in Wnt signaling. We also relate its expression to the androgen receptor and MMP-7 protein, both critical to prostate cancer pathogenesis. Some mutations in the androgen receptor also impact the androgen-β-catenin axis and hence, lead to the progression of prostate cancer. We have also reviewed MiRNAs that modulate this pathway in prostate cancer. Finally, we have summarized the impact of Wnt/β-catenin pathway proteins in the drug resistance of prostate cancer as it is a challenging facet of therapy development due to the complexity of signaling pathways interaction and cross-talk.

## Introduction

Wnt signaling cascade is a conserved pathway, which is crucial for the determination of cell fate and embryonic patterning in all multicellular organisms [1]. Due to its initial discovery in the wingless mutant of Drosophila, it was first named *Wingless* [2]*.* Later, a homolog of this gene in the integration region or *int* was identified in the mouse, where proviral sequence insertion at 5′ or 3′ end caused tumorigenesis in the mammary tissue [3]. Due to the homologous nature of both sequences, names were combined as *Wnt* [4]*.* This gene is involved in a wide range of cellular processes like cell multiplication, migration, apoptosis, differentiation, etc., and regulates several other genes [5]. Extracellular regulation of the signaling process is carried out by a complex pathway, involving an ensemble of antagonists, co-receptors, and co-factors. Around 19 such ligands bind 10 Frizzled family G-protein-coupled receptors for activity [1]. The key modulator of Wnt signaling i.e., β-catenin is controlled by a cluster of complex proteins in the cytoplasm whereas, in the nucleus, essential gene activation is carried out by the reciprocated activity of repressors, activators, and other proteins. Wnt proteins are made up of 22 cysteines as well as one or more N-linked glycosylation sites [6].

**Fig. 1:**
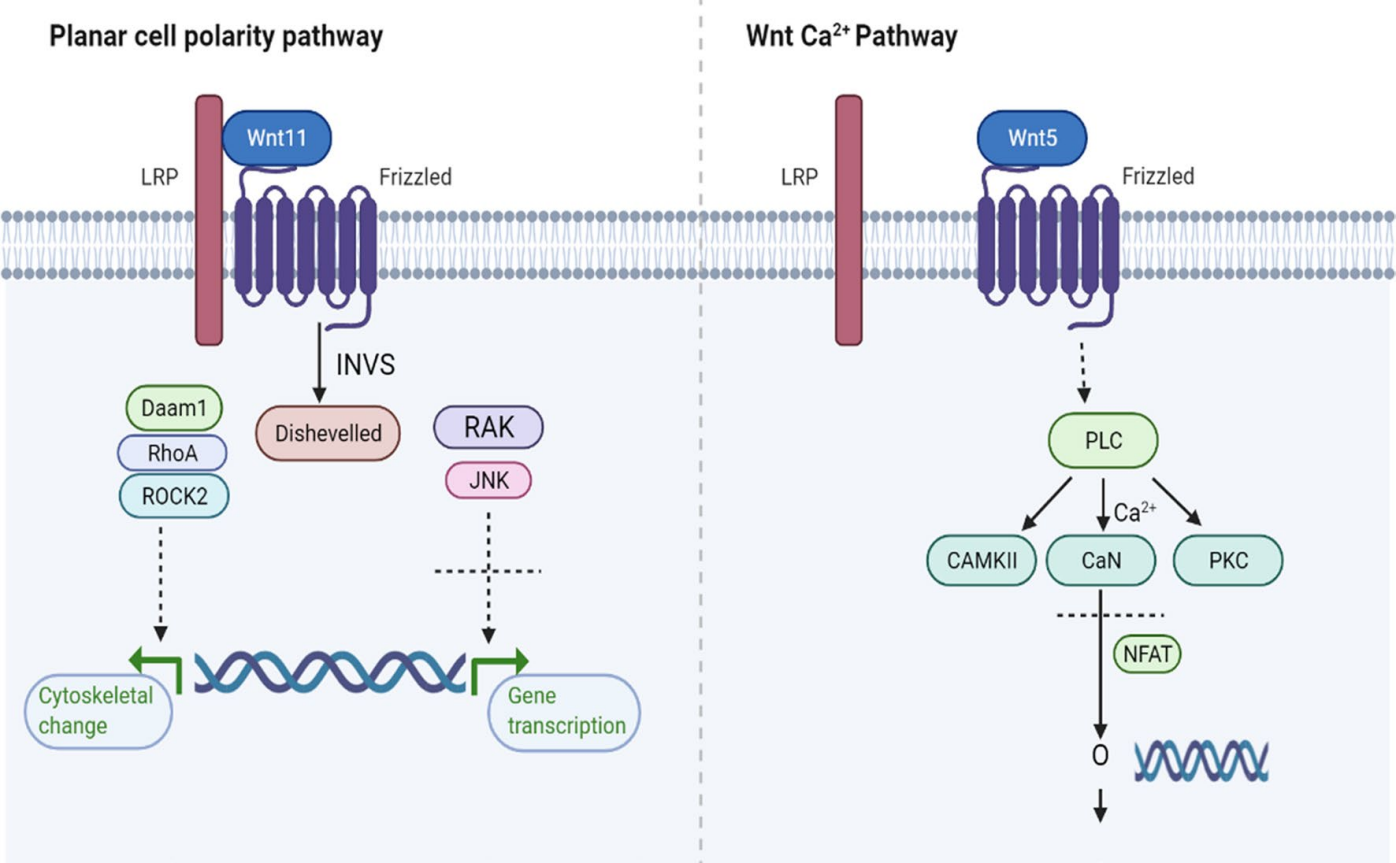
Planar cell polarity and Wnt/Ca^2+^ pathway. Figure adapted from KEGG database

The initial activation step of this pathway involves a specific Wnt ligand binding to its specific Frz group proteins, and one of the three branch reactions is initiated depending on the signal (Fig. 1). If the Wnt signal is absent, β-catenin is not released from the GSK3b/APC/axin complex grip and subsequently, it degrades. If a signal is present, Dvl/Frat protein complex inactivates GSK3b. This leads to β-catenin buildup due to disruption of the quaternary complex. The branch reaction pathways initiated as result, impact each other as well as act as part of other signaling networks [7]. Pathway activation is controlled by the specific Wnt/Frz combination (with or without certain co-receptors), and thereafter, diversification occurs via protein–protein interactions. The branching pathway determines many cell-fate decisions and can be divided into the canonical and non-canonical pathway. These pathways are not entirely independent of each other and do overlap during several cellular processes [4]. Previously it was thought that the varied phosphorylation profile of Dvl protein was the reason for this pathway dichotomy but later Grumolato et al*.* [8] revealed that the Wnt signal transduction outcome is based on the ligand binding with shared, allied receptors and unrelated co-receptors. They discovered that the canonical pathway was activated in the presence of and non-canonical in the presence of Wnt5a. When non-canonical was activated, phosphorylation of co-receptors LRP5/6 and Ror2, arbitrated by GSK also occurred. LRP6 binds with Dkk proteins which antagonize the Wnt/β-catenin pathway while Ror2 receptors undergo phosphorylation in the presence of Wnt5 [9]. Information about several other receptors in non-canonical pathway is still lacking but a combination of factors involving receptor-ligand interaction is involved. Another type of Wnt pathway exists in *C. elegans* and *D. melanogaster,* which controls asymmetric cell division and spindle orientation [10–12] but we will not discuss it as it is not relevant to the topic of interest.

## Non-canonical pathway

Non-canonical pathway further branches into two types i.e., Wnt/Ca^2+^ and planar cell polarity (PCP) pathway. These pathways do not require β-catenin as a co-transcription factor and are known to be initiated by several Wnt ligands, depending on the context. Among these, Wnt3a regulates cell differentiation [13] while Wnt4 regulates cell expansion [14], migration [15], cell proliferation, and tumor growth [16] in different scenarios. Wnt5a/b and Wnt11 activate the pathway in cell polarization and migration [17, 18], while Wnt6 controls migration and differentiation [19]. Wnt/Ca^2+^ pathway regulates cell adhesion as well as motility [20]. The cycle involves activation of dishevelled (Dsh) phospholipase C, which in turn raises the level of Ca^2+.^ This leads to the activation of calcium-sensitive protein kinase C and calmodulin-dependent protein kinase (CAMKII). High Ca^2+^ levels cause the phosphatase calcineurin activation, which causes dephosphorylation of transcription factor NF-AT. NF-AT is then accumulated in the nucleus, which further leads to target gene activation [21, 22]. Wnt ligands may also be aided by the co-receptors and bind to Frz proteins, for C-Jun N-terminal kinase (JNK) activation via an activator protein. Wnt/Frz interaction turns the small GTPases machinery on, inducing polarization and cytoskeletal rearrangements of the cell. The planar cell polarity pathway controls the cell polarity and morphogenetic movements in a plane of tissue. The process occurs via activation of actomyosin and is arbitrated by the activity of small GTPases (RhoA/B, Rac1, Cdc42) and JNK [12, 23].

## Canonical Wnt or β-catenin pathway

**Fig. 2:**
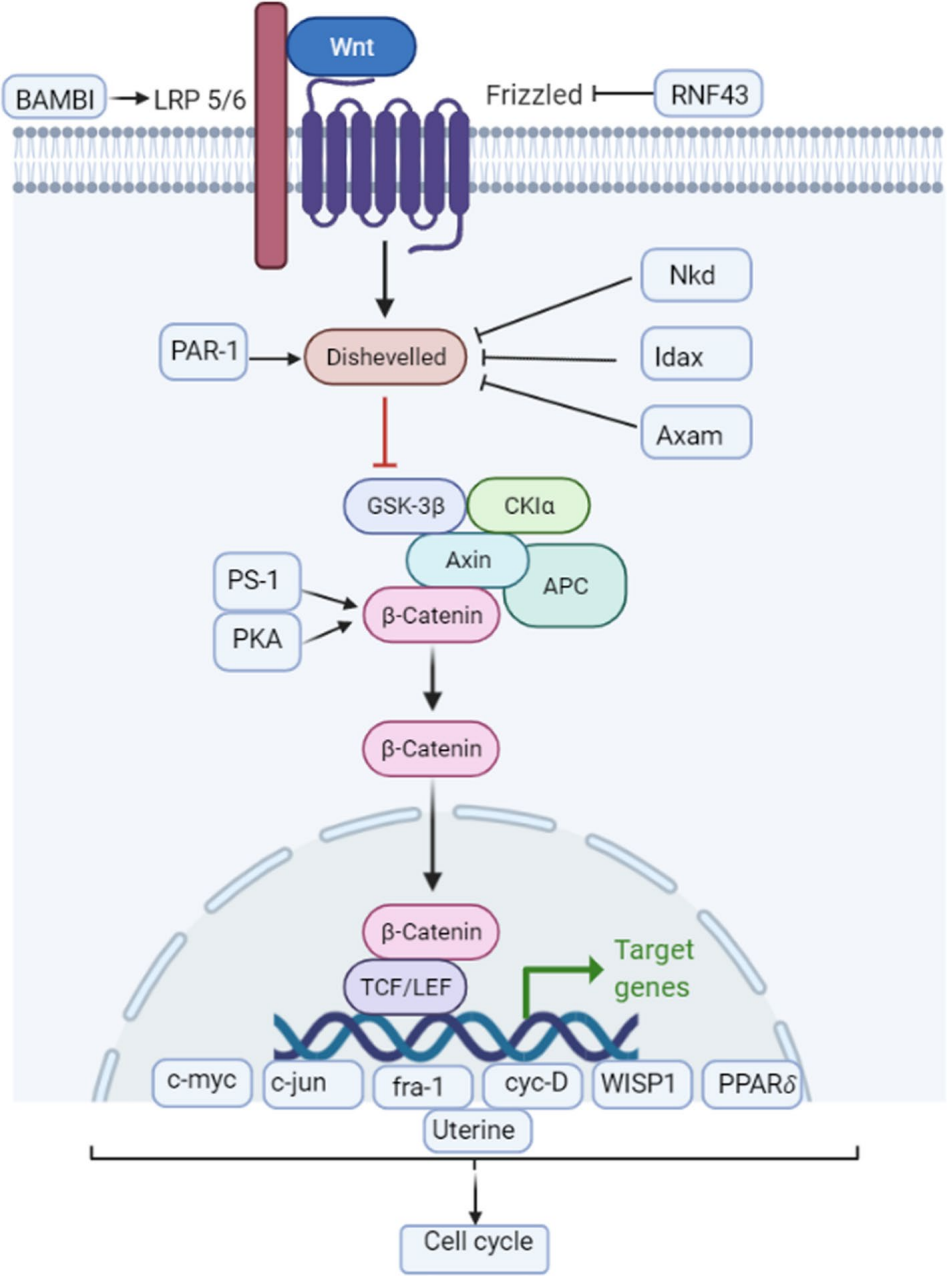
Canonical Wnt/β-catenin pathway. Figure adapted from KEGG database

Canonical Wnt or β-catenin pathway (Fig. 2) is better understood compared to the non-canonical pathways. This pathway mediates cell proliferation as well as differentiation, with β-catenin as the major player. Signaling is initiated by the Wnt 1, 2, 2b, 3, 3a, 4, 5a/b, 6, 7a/b, 10a/b, 11 and 16 whereas Wnt-specific targets are activated by the β-catenin/T-cell transcription factor [1, 24, 25]. In the absence of the Wnt signal, β-catenin levels remain low in the cytoplasm due to a coordinated action of Axin, Casein kinase 1a (CK1a) and Glycogen synthase kinase 3b (GSK3b) protein complex. Axin and APC provide structural support to β-catenin. CK1a and GSK3b phosphorylate it at the N-terminal end. It binds E3 ubiquitin ligase and represses Wnt target genes due to the impact of DNA bound transcription factor [T-cell factor/lymphoid enhancer-binding factor (TCF/LEF)] bonding with Groucho. For accumulation of β-catenin in the cytoplasm, leading to higher concentration, this degradation complex is silenced [1, 5]. The mechanism involves axin binding of phosphorylated lipoprotein receptor-related protein (LRP) tail and polymerized Dsh. Β-catenin increases in the cytoplasm and is also transported to the nucleus, eventually replacing Groucho in the later stage. TCF/LEF is then activated and Fzr related proteins are released along with the secretion of the Wnt inhibitory factor [1]. These can inhibit the pathway in addition to the non-canonical signaling elements (specifically Wnt5a and Wnt11), which inhibit in a Ca^2+^ dependent and independent way [26, 27].

Wnt signaling is critical for β-catenin stability while the conventional role of β-catenin was to modulate cadherin-arbitrated intercellular adhesion [28, 29]. Wnt signaling also curbs the β-catenin through ubiquitination mediated degradation [30]. Conventionally, β-catenin is phosphorylated in the cytoplasm and is prone to proteasomal mediated degradation after binding the APC/GSK3β/axin complex. However, when the Wnt signal is present, β-catenin remains unphosphorylated and subsequently accumulates in the cytoplasm [24].

## Wnt/β-catenin pathway relation to prostate cancer

The wnt/β-catenin pathway is well studied and has a role in many cancers [31–35]. The wnt/β-catenin pathway is regulated at multiple levels: gene mutations, extracellular inhibitors, and intranuclear transcription co-factors whereas its dysregulation leads to cancer. Higher levels in the cytoplasm and nucleus target intended genes, for instance, cyclic D1 and c-myc. We focus on Wnt/β-catenin relation to prostate cancer in this article. The Wnt gene was demarcated as an oncogene by the scientists but it was discovered later that not all Wnt genes were capable of inducing cancer. Foxa2, a downstream target induced by Wnt β-catenin, is important for the establishment of prostate cancer bone metastasis [36]. Moparthi et al. [37] recorded that Foxb2, which acts as a tissue-specific Wnt activator might also play a role in prostate cancer progression. In addition, the mutations in β-catenin, which intertwines with the androgen receptor pathway, are responsible for prostate cancer. Voeller et al. [38] using single-strand conformation polymorphism (SSCP), discerned five mutations in the regulatory site in a panel of more than a hundred prostate cancers. These mutations influenced late tumorigenesis where four were linked to phosphorylation sites and one impacted a residue adjacent to Ser33.

Wingless-related MMTV integration site 1 or Wnt-1 occurs in shallow quantity in primary prostate epithelial cells but it has been observed that it is up-regulated in prostate cancer cell lines and tissues, especially in the lymph node and bone metastases samples [39]. Similar observations have been recorded by de la Taille et al. [40] for metastatic and hormone-refractory prostate carcinoma [41]. Thiel et al. [42] also recorded high levels of Wnt-1 in DU145, which is a human prostate cancer cell line. Katoh [43] reported high Wnt-2 in primary prostate cancer tumors. Hall et al. [44] reported enhanced Wnt-2 in prostate cancer metastases compared to the primary lesions. It has been noted that Wnt3a can impact the progression of prostate cancer by enhancing the expression of cytosolic and nuclear β-catenin in addition to enhancing androgen receptor activity or inducing activity in absence of the androgen hormone [45, 46]. Wnt5a acts as a tumor suppressor gene for several cancers but a protooncogene for prostate cancer. It has a varied methylation profile of CpG island in 5′ UTR and epigenetic changes regulate the expression. However, Zhao et al. [47] demonstrated via miR-26a repression of Wnt5a that this Wnt is involved in prostate cancer progression. Yokoyama et al. [48] and Yamamoto et al. [49] have shown that due to activated JNK and matrix metalloproteinase-1 (MMP-1), expression of Wnt5a is elevated, which assists invasion of cells and leads to prostate cancer. Wnt-6 is enhanced in prostate cancer impacted tissues versus normal ones [50, 51]. WNT11 expression has also been found to be enhanced in the prostatic tumors of higher grade as well as in the hormone-independent prostate cancer cell lines [52]. Wnt-7B [53, 54] and Wnt-13 are usually expressed in normal prostate tissues [45, 55]. Wnt-10b is downregulated in localized prostate cancer specimens compared to benign tissue [56]. Genomic alterations or mutations in the Wnt/β-catenin pathway leading to prostate cancer have been reviewed in detail elsewhere [57].

## β-catenin and androgen receptor cross-talk in prostate cancer

Some researchers have also implied the role of androgen receptor in the progression of prostate cancer [58]. A downstream effector of the β-catenin pathway was shown to be an androgen receptor co-activator, which is an oncogene and implicated in prostate cancer. Peptidyl-prolyl isomerase Pin1 can hinder androgen receptor interaction with the β-catenin and modulate prostate cancer cell proliferation [59]. Seo et al. [60] reported that in the absence of androgens, which are causative of prostate cancer as well, the Wnt/β-catenin pathway up-regulates Yes-associated protein (YAP) from the Hippo pathway and switches on the androgen arbitrated transcription pathway. Wnt3a enhances the androgen receptor and YAP protein levels and increases their nuclear translocation. Inhibition of androgen activity leads to enhanced expression of the Wnt/β-catenin pathway, which in turn promotes androgen-independent growth of prostate cancer cells. When androgen receptor is missing, β-catenin recruitment to TCF binding sites results in increased transcription and hence, cellular proliferation. It has been implicated in some researches that β-catenin in prostate cancer does not merely activate TCF/LEF transcriptionally, but it also leads to androgen sensitivity. SOX9 transcription factor also impacts β-catenin and androgen receptor, leading to invasive prostate cancer [61]. Androgen mutations lead to androgen-independent prostate cancer progression by activating the Wnt/β-catenin pathway. These are rare in the early stages of cancer but have an enhanced frequency in late-stage tumors [62–64]. Around nine deletion (Table 1) and more than 70 different somatic missense androgen receptor mutations have been described in patients with prostate cancer, with several having gain of function [64]. The number has now increased to more than 1000.

**Table 1:** Deletion mutations in the androgen receptor, responsible for androgen-independent prostate cancer. Mutations were retrieved from the Androgen receptor mutation database (URL: http://androgendb.mcgill.ca/)

Serial No.	Position	Details	References
1	58–80	Contraction of poly Gln repeats (24 to 18)	[65]
2	58–80	Deletion of 1 poly Gln repeat (23 to 22)	[66]
3	58–80	Contraction of poly Gln repeats (20 to 18)	[37]
4	086	Gln86 deletion	[67]
5	548	Leu547 frameshift	[68]
6	555	Pro555 frameshift	[68]
7	555	Pro 555 frameshift	[68]
8	744	Gly 744 frameshift	[68]
9	748	Phe748 frameshift	[69]

In conventional conditions, Wnt/β-catenin and androgen signaling does not cross pathways and works differently in different cells but in cancer, it affects androgen signaling, especially during the androgen-independent prostate cancer signaling. It allies with the androgen receptor and translocates in the presence of androgens, from the cytoplasm to the nucleus, enhancing transcription of the androgen receptor. This has been demonstrated by yeast-two hybrid assays [70]. However, immunofluorescence assays that utilized anti-β-catenin and anti-androgen receptor antibodies have demonstrated that β-catenin and androgen receptor primarily present in the cytoplasm during in-activated Wnt cycle. β-catenin suppression via GSK3 also increases androgen receptor activity and vice versa. Insulin-like growth factor (IGF) induces androgen receptor signaling via β-catenin, leading to the proliferation of prostate cancer. PI3K/AKT and PTEN also enhance androgen-mediated transcription, leading to increased prostate cancer cell survival and growth. Androgen receptor may also attach with LEF transcription factor, influencing β-catenin and prostate cancer progression [71].

Jung et al. [72] also studied the cytoplasmic β-catenin expression at various stages of human prostate cancer along with bone metastasis and preoperative prostate-specific antigen (PSA) level. Cytoplasmic β-catenin expression ranged from moderate to strong in less than 20% of localized prostate cancer. It was around 35.5% in locally advanced prostate cancer and 60% in metastatic prostate cancer. This shows a definitive role of the β-catenin pathway and its cross-talk with androgen receptor in the progression of prostate cancer.

## β-catenin and MMP-7 cross-talk in prostate cancer

In addition to involvement in cross-talk with the androgen receptor, β-catenin also has a crucial role in the expression of matrix metalloproteinase MMP-7, which also alters cancer cell characteristics. MMP-7 is part of the protein ensemble which breaks down the extracellular matrix. Breaking of the external matrix is pivotal for tumor invasion and metastasis. Wnt/β-catenin pathway controls MMP-7 in a way that its accumulation leads to the MMP-7 overexpression [72]. Grindel et al. [73] reported similar findings that a proteoglycan expressed in the membrane underlying epithelial and endothelial cells were lysed by MMP-7. Hence, it plays the role of a molecular switch and aids cell dispersion in an invasive tumor microenvironment. Reid et al. [74] have demonstrated both in vitro and in vivo that aberrant expression of MMP is linked with invasion and metastasis of prostate tumors. β-catenin, MMP-7, and androgen receptor in the cytoplasm have been tracked down as positively correlated. Furthermore, the expression of these three genes is also significantly correlated with clinicopathological characteristics of prostate cancer [72]. This shows that the intracellular Wnt/β-catenin signaling is interactive and can impact prostate cancer progression with and without the aid of other genes like androgen receptor and MMP-7. Hashimoto et al. [75] previously reported that the MMP-7 mRNA level is enhanced in pathological stages, of prostate cancer. Zhang et al. [76] reported that MMP7 advances prostate cancer by instigating epithelial-to-mesenchymal transition. In mouse models, MMP-7 enhanced the expression of EMT transcription factors by breaking the E-cadherin/β-catenin complex.

## Other Wnt/β-catenin linked factors with a role in prostate cancer

ETS-related gene (ERG) upregulates the downstream mediator transcription factor of the Wnt/β-catenin signaling pathway named Lymphoid enhancer-binding factor 1 (LEF1), which in turn enhances the androgen receptor expression and activity, leading to prostate cancer in an androgen-independent manner [77]. Cadherins also cross-talk with the Wnt signaling pathway where E-cadherin expression is decreased and N-cadherin is increased in multiple prostate cancer cell lines. The decreased expression indicates metastasis and enhanced expression is allied with cellular migration, invasion, and shorter cancer recurrence [78]. A cadherin switch (formed upon the suppression of E-cadherin and overexpression of N-cadherin) is involved in prostate cancer progression where E-cadherin obstruction by LEF1 and support of N-cadherin permit the cells to undergo epithelial-to-mesenchymal transition [77].

Mutations in other components of the Wnt Pathway have also been linked with prostate cancer. These include inactivating mutations in the two genes negatively regulating the Wnt pathway. These genes transcribe E3 ubiquitin-protein ligases: Ring finger 43 (RNF43) and Zinc And Ring Finger 3 (ZNRF3) ligase [79]. Antagonists of both these genes, R-Spondin 2 (RSPO2) and R-Spondin 3 (RSPO3) fusions potentiate the Wnt/β-catenin signaling and have been linked with prostate cancer [80]. Loss of expression of the genes WNT Inhibitory Factor 1 (WIF1) and Secreted Frizzled Related Protein 1 (sFRP1), due to promotor methylation inhibits Wnt signaling and is associated with prostate cancer [29, 48, 81].

Liu et al. [82] reported that Cripto-1 promotes epithelial-mesenchymal transition in prostate cancer through Wnt/β-catenin signaling. Seo et al. [60] reported that Wnt/β-catenin also interacts with the Hippo pathway protein YAP, where Wnt3a promotes prostate cancer cell growth in an androgen-independent manner. Cheng et al. [83] reported YAP1 expression elevation in high-grade prostate adenocarcinomas. They also assessed the impact of YAP1 in neuroendocrine prostate cancer mice models. Wnt7A expression was increased. Expression of the DKK1, which is an inhibitor of Wnt [84] was decreased in wild-type prostate but not in transgenic adenocarcinoma of the mouse prostate. It has also been implicated that the Ras signaling pathway intertwines with the Wnt pathway at some points and aids bone metastasis in prostate cancer [85].

There has been evidence of some micro-RNAs (miRs) modulating prostate cancer by regulating the Wnt/β-catenin signaling pathway. miR-15A [86], miR-133A, miR-133B [87], miR-320 [88] downregulate in prostate cancer and influence Wnt signalling. miR‐138 affects cell invasion and relocation of prostate cancer cells through the Wnt pathway [89]. miR-939 deactivates the Wnt path by controlling hepatoma-derived growth factor (HDGF) and modulates prostate cancer [90]. miR-744 [91], miR-182 [92] activates the Wnt/β-catenin pathway and promotes prostate cancer. MiR-21 promotes by impact Wnt11 [93]. MiR-26a inhibits the progression of this cancer by suppressing Wnt5a [47] while hsa-miR-1297 downregulates Wnt/AEG1 pathway signaling [94]. Several other miRs impact prostate cancer progression by either suppressing or promoting genes in the Wnt/β-catenin signaling pathway directly or indirectly [95, 96]. Androgen receptor is also impacted by several miRNAs, which lead to up or downregulation of prostate cancer (Table 2), which indirectly influences the Wnt/β-catenin axis.

**Table 2:** miRNAs impacting androgen and probable Wnt-β-catenin axis in prostate cancer. These are manually curated and retrieved from http://mircancer.ecu.edu/search.jsp

Serial No.	miRNA	Up or down regulation	References
1	let-7c	Down	[97]
2	miR-27	Down	[98]
3	miR-34b	Down	[99]
4	miR-135a	Down	[100]
5	miR-145	Down	[101]
6	miR-182	Up	[92]
7	miR-187	Down	[102]
8	miR-212	Down	[103]
9	miR-221	Up	[104]
10	miR-331	Down	[105]
11	miR-421	Down	[106]

## Drug resistance in prostate cancer

Several drugs exist that target the Wnt/β-catenin signaling in prostate cancer and possibly alleviate the suffering of patients, afflicted by this terrible disease. Drugs of choice are small molecule inhibitors because of their efficiency to pierce membranes and reach inner cells for disrupting the function. These have been reviewed elsewhere and include transcription factors, interactors, and ligands of the pathway [107]. A list of approved drugs for prostate cancer is given at the National cancer website of the USA (https://www.cancer.gov/about-cancer/treatment/drugs/prostate) while several of them are in clinical trials [29, 81, 108]. Resistance to several of these drugs occurs in patients, rendering the treatment useless and resulting in loss of life. Although several reviewers have been conducted on drug resistance in prostate cancer [109–112], we focus on Wnt/β-catenin pathway elements conferring resistance to drug or therapy in prostate cancer. It is pertinent to mention that localized cancer is treated with surgery and radiation whereas advanced-stage cancer is treated with hormone therapy. Usually, the Wnt/β-catenin pathway is activated at end-stage of the prostate cancer and promotes drug resistance [107]. This end-stage activation is due to interaction with several moieties like cytokines/growth factors from the tumor, inhibition of androgen receptor and β-catenin release along with attachment, alteration, and modulation of pathway ligands. Chemotherapy or radiation promotes Wnt signaling and protects proliferating cells from cell cycle arrest or apoptosis [113]. Since Wnt/β-catenin elements cross-talk with the androgen pathway, β-catenin signaling also confers resistance to androgen inhibitors. Therapy targetting both Wnt/β-catenin and androgen would be better in such cases.

It has been observed in gene expression studies that Wnt/β-catenin signaling was present in enzalutamide-resistant tumors whereas patients lacking the active pathway were found to be sensitive to the treatment [114]. Enzalutamide is an androgen suppressor and combined targetting of androgen receptor and Wnt/β-catenin represses cell growth in both androgen-dependent and -independent manner [115]. Cristobal et al. [116] reported that upregulation of CIP2A/Plk1 and androgen/Wnt/β-catenin axis could contribute to taxane-based therapy resistance in prostate cancer cells via mitosis-related proteins. Chen et al. [117] revealed that missense mutations in the β-catenin gene were associated with enzalutamide resistance.

Kohli et al. [118] proved through a genome-wide study that abiraterone acetate therapy is rendered useless by Wnt pathway elements. Wang et al. [92] demonstrated that a large number of patients were resistant to abiraterone-acetate-prednisone therapy resistance due to mutations in the Wnt/β-catenin pathway. Patients conferring resistance to the therapy had a higher frequency of downregulated Wnt/β-catenin genes. The indirect impact of Wnt/β-catenin signaling in cisplatin resistance has been observed by enhancing the expression of ABC transporter and mediating efflux of the drug. Wnt7b impacts two such genes i.e. ABCB1 and ABCG2 which are responsible for drug efflux [119]. ABCB1 has been shown to confer resistance to docetaxel, which leads to cross-resistance to cabazitaxel [120]. Zhu et al. [121] found that caspiacin activity on prostate cancer, Bian et al. [122] reported that Wnt/β-Catenin signaling is activated in docetaxel-resistant hormone-refractory prostate cancer cells, which can be promoted by E3 ubiquitin ligase. Apart stem cells can be suppressed by the activation of Wnt/β-catenin signaling. Recently from drugs, radiation resistance has also been conferred by the activation of ALDH1A1 due to Wnt/β-catenin signaling in prostate cancer patients. Blocking the pathway led to the resensitization of the patients to radiation therapy [123].

## Conclusion

Prostate cancer is an ailment of the male reproductive system. Canonical and non-canonical Wnt pathway mediates this hormone-sensitive disease where surgery and radiation do not often work. Wnt/β-catenin has a pivotal role in prostate cancer pathogenesis, proliferation, and resistance to treatment. We have reviewed various aspects of this pathway signaling in prostate cancer and drug resistance modulated by these pathway elements. Patients undergo treatments like androgen deprivation along with blockage of interacting moieties like Wnt/β-catenin elements. Many patients do not respond to even these therapies because of alterations of a corresponding pathway or gene cross-talk of other pathways with Wnt elements. Deeper mechanistic studies need to be conducted on this pathway mediation of prostate cancer and drug resistance in it as full-fledged knowledge of these pathway interactions with prostate cancer cells is essential for devising effective therapeutic strategies and avoiding resistance to the drugs.
